# Expression of steroidogenic enzymes and metabolism of steroids in COS-7 cells known as non-steroidogenic cells

**DOI:** 10.1038/s41598-018-20226-2

**Published:** 2018-02-01

**Authors:** Mitsuki Nozaki, Shogo Haraguchi, Takuro Miyazaki, Daichi Shigeta, Noriko Kano, Xiao-Feng Lei, Joo-ri Kim-Kaneyama, Hiroyuki Minakata, Akira Miyazaki, Kazuyoshi Tsutsui

**Affiliations:** 1 Laboratory of Integrative Brain Sciences, Department of Biology and Center for Advanced Biomedical Sciences of Waseda University, Tokyo, 162-8480 Japan; 2 Department of Biochemistry, Showa University School of Medicine, Tokyo, 142-8555 Japan; 3 Suntory Foundation for Life Sciences, Kyoto, 619-0284 Japan

## Abstract

The COS-7 (CV-1 in Origin with SV40 genes) cells are known as non-steroidogenic cells because they are derived from kidney cells and the kidney is defined as a non-steroidogenic organ. Therefore, COS-7 cells are used for transfection experiments to analyze the actions of functional molecules including steroids. However, a preliminary study suggested that COS-7 cells metabolize [^3^H]testosterone to [^3^H]androstenedione. These results suggest that COS-7 cells are able to metabolize steroids. Therefore, the present study investigated the expression of steroidogenic enzymes and the metabolism of steroids in COS-7 cells. RT-PCR analyses demonstrated the expressions of several kinds of steroidogenic enzymes, such as cytochrome P450 side-chain cleavage enzyme, 3β-hydroxysteroid dehydrogenase/Δ^5^-Δ^4^ isomerase, cytochrome P450 7α-hydroxylase, cytochrome P450 17α-hydroxylase/17,20-lyase, 17β-hydroxysteroid dehydrogenase, 5α-reductase, cytochrome P450 21-hydroxylase, cytochrome P450 11β-hydroxylase, and cytochrome P450 aromatase in COS-7 cells. In addition, steroidogenic enzymes 3β-HSD, P4507α, 5α-reductase, P450c17, P450c21, P450c11β, and 17β-HSD actively metabolized various steroids in cultured COS-7 cells. Finally, we demonstrated that 17β-HSD activity toward androstenedione formation was greater than other steroidogenic enzyme activities. Our results provide new evidence that COS-7 cells express a series of steroidogenic enzyme mRNAs and actively metabolize a variety of steroids.

## Introduction

The COS-7 (CV-1 in Origin with SV40 genes) cell line was developed by Prof. Yakov Gluzman in the early 1980s. It is derived from the CV-1 African green monkey kidney fibroblast cell line transformed by a mutant strain of Simian Virus 40 (SV40) that codes for the wild-type T-antigen^1,2^. This cell line has unique characteristics of fibroblast-like growth and virus susceptibility^1,2^. These characteristics make COS-7 cells a popular research tool and an excellent choice for DNA plasmid transfection experiments^1–5^.

Many previous studies have reported that COS-7 cells are non-steroidogenic cells^6–8^. The COS-7 cell line is derived from kidney cells and the kidney is defined as a non-steroidogenic organ^9,10^. Therefore, COS-7 cells have been used for transfection experiments to analyze the functions of steroidogenic genes^11–13^, steroid receptors^14–16^, and the effects of steroids on functional molecules^17,18^.

A preliminary study in our laboratory suggested that COS-7 cells actively metabolize [^3^H]testosterone to [^3^H]androstenedione (S. Haraguchi *et al*., unpublished observations). In addition, the expression of steroidogenic enzymes in the kidney of humans^19–21^ and rodents^22–24^ has been reported. These results suggest that COS-7 cells may metabolize steroids.

Based on this background, in the present study, a series of experiments was conducted to demonstrate the expression of steroidogenic enzymes and metabolism of steroids in COS-7 cells, which are known as non-steroidogenic cells. Because pregnenolone formation is the first step in steroid synthesis^25–27^, we first investigated the formation of [^3^H]pregnenolone from [^3^H]cholesterol in cultured COS-7 cells. Steroidogenic acute regulatory protein (StAR; gene name *Star*) delivers cholesterol to the mitochondrial cytochrome P450 side-chain cleavage enzyme (P450scc; gene name *Cyp11a*), which produces pregnenolone. RT-PCR analyses demonstrated P450scc mRNA expression in COS-7 cells. We further demonstrated that the mRNAs of several kinds of steroidogenic enzymes 3β-hydroxysteroid dehydrogenase/Δ^5^-Δ^4^ isomerase (3β-HSD; gene name *Hsd3b*), cytochrome P450 7α-hydroxylase (P4507α; gene name *Cyp7b*), cytochrome P450 17α-hydroxylase/17,20-lyase (P450c17; gene name *Cyp17*), 17β-hydroxysteroid dehydrogenase (17β-HSD; gene name *Hsd17b*), 5α-reductase (gene name *Srd5a*), cytochrome P450 21-hydroxylase (P450c21; gene name *Cyp21*), cytochrome P450 11β-hydroxylase (P450c11β; gene name *Cyp11b1*), and cytochrome P450 aromatase (P450arom; gene name *Cyp19*) are expressed in COS-7 cells. In addition, steroidogenic enzymes 3β-HSD, P4507α, 5α-reductase, P450c17, P450c21, P450c11β, and 17β-HSD were active in cultured COS-7 cells. Especially, androstenedione formation from testosterone catalyzed by 17β-HSD was greater than other steroidogenic enzyme activities. Our results provide new evidence that COS-7 cells express a series of steroidogenic enzyme mRNAs and actively metabolize a variety of steroids.

## Materials and Methods

### COS-7 cells

The COS-7 cell line (JCRB9127) was purchased from the Japanese Collection of Research Bioresources (JCRB) cell bank (Osaka, Japan). COS-7 cells were maintained in DMEM (043-30085; Wako, Osaka, Japan) supplemented with 10% FBS (S1820-500; BioWest, Nuaill, France) and a 1% penicillin-streptomycin solution (Wako, Osaka, Japan) at 37 °C in a humidified 5% CO_2_-containing atmosphere. COS-7 cells were used in the experiments between passages 3 and 15, or passages 30 and 40.

### RT-PCR analyses of steroidogenic enzyme mRNAs

Total RNA was extracted from COS-7 cells with Sepazol-RNA I Super (Nacalai Tesque, Kyoto, Japan) and treated with RNase-free DNase I (Wako, Osaka, Japan), then reverse-transcribed with M-MLV reverse transcriptase (Promega, Madison, WI, USA) according to the product instructions. All PCR amplifications (for StAR, P450scc, P4507α, 3β-HSDs, 5α-reductases, P450c17, P450c21, P450c11β, 17β-HSDs, and P450arom) were performed in a reaction mixture containing *Ex Taq* polymerase^13,28–30^ (Takara, Shiga, Japan). Forward and reverse primers (Table 1) were designed according to the nucleotide sequence of African green monkey steroidogenic enzyme mRNAs. The following PCR conditions were used on the thermal cycler: 1 cycle of 1 min at 94 °C, 30 cycles of 30 s at 94 °C, 30 s at 60 °C, 30 s at 72 °C, and finally, 1 cycle of 10 min at 72 °C. The identities of the PCR products were confirmed by sequencing. COS-7 cells were used in the experiments between passages 3 and 15.

**Table 1:** Primers for PCR analyses.

Primer	Forward primer 5′->3′	Reverse primer 5′->3′
StAR	GAGCTCTCTGCTCGGTTCTC	CGCCCTGATGACACCTTTCT
P450scc	CCCGATTTACAGGAAGTCAGC	TCTGTAGAGGATGCCACGGT
3β-HSD type I	GAGGGAGGAATTTTCCAAGCTCCA	GTCTTTCAGAGTCCACCCATCA
3β-HSD type VII	CCTTCTACAGGGGCAACGAA	GAACACCAGCAGCCAGTAGG
P4507α	GCCTGATCTGCCTAGAAAGCA	TGAATACCAAACAACAAGCGGT
P450c17	CGGCCTCAAGTGACAACTCT	GGTGATAGAGTCACTGCGGAA
17β-HSD type I	TCAGACCCATCCCAGAGCTT	CTCGATCAGGCTCAAGTGGAC
17β-HSD type II	CAAATGGACGTCACGAAGCC	CGTGCCTGCGATACTTGTTC
17β-HSD type III	CCAAAGCCTTTCTTGCGGTC	CACACAAACGCCTTGGAAGC
17β-HSD type IV	TGGCCAGCTATGATTCAGTGG	CAAAGCCAAAGGACAAGCGG
5α-Reductase type I	CGGGCATCGGTGCTTAATTT	GAGTGCATGACAGCAGGAGA
5α-Reductase type II	GTGCATTACTTCCACAGGACA	CAGCCCAAGGAAACAAACCG
5α-Reductase type III	TCAGTGCTGTGGAATGGCTT	CAGTGAATGACCACTCCTGCT
P450arom	TCCCTTTGGACGAAAGTGCT	CTGGTACCGCATGCTCTCAT
P450c11β	GATAGCCTGCATCCCCACAG	AGTTGTCGCCGTACTGGAAG
P450c21	CGACCTCCCCATCTATCTGC	GGGAAGAACTTGATCTTGTCTCCA

### Western blot analyses of steroidogenic enzyme proteins

Western blot analyses were performed on the proteins of COS-7 cells as described previously^13,25–30^. The protein of COS-7 cells were separated on a 12.5% SDS-polyacrylamide gel under reducing conditions and transferred to PVDF membranes (Hybond-P; GE Healthcare, Little Chalfont, UK). The membranes were incubated with mouse anti-5α-reductase type I antibody (66329-1-lg; Proteintech, Chicago, IL, USA), rabbit anti-17β-HSD type II antibody (10978-1-AP; Proteintech), or rabbit anti-17β-HSD type IV antibody (15116-1-AP; Proteintech) at 4 °C overnight and then for 1 h with anti-mouse IgG, HRP-linked antibody (#7076; Cell Signaling Technology, Beverly, MA) diluted 1:2,000 or anti-rabbit IgG, HRP-linked antibody (#7074; Cell Signaling Technology) diluted 1:2,000. Intense immunoreactive bands were detected by using ImmunoStar Zeta detection kit (Wako). COS-7 cells were used in the experiments between passages 3 and 15.

### Biochemical analysis of cholesterol metabolism

To investigate cholesterol metabolism, COS-7 cells were incubated with [^3^H]cholesterol and the radioactive metabolites were analyzed by reversed-phase HPLC. Biochemical analysis was performed as described previously^13,25–30^. In brief, COS-7 cells were plated in 10 cm^2^ culture dishes and grown in DMEM supplemented with 10% FBS to 90% confluence. Cells were then cultured in serum-free DMEM containing 70 nmol [^3^H]cholesterol (specific activity, 53.0 Ci/mmol; PerkinElmer, Waltham, MA, USA) for 0 or 6 h at 37 °C in a water-saturated atmosphere (5% CO_2_, 95% air) to maintain the pH at 7.4. After incubation, steroids were extracted with ethyl acetate and subjected to HPLC analysis using the reversed-phase column, Capcell Pak C18 MG (Shiseido, Tokyo, Japan). HPLC was performed with an isocratic condition of acetonitrile/isopropanol (60:40, vol/vol) at a flow rate of 0.3 ml/min. Eluted fractions were collected every 30 s from 0 to 30 min and counted in a liquid scintillation counter (Tri-Carb 2810TR; PerkinElmer). A reference standard of tritiated cholesterol was chromatographed to detect its elution position.

### Biochemical analyses of steroids formed from pregnenolone

To investigate steroid formation from pregnenolone in COS-7 cells, conversions of substrate steroids (pregnenolone, progesterone, androstenedione, or testosterone) were measured biochemically as described previously^13,25–30^. In brief, at 90% confluence, COS-7 cells were cultured in serum-free DMEM containing 70 nmol [^3^H]pregnenolone (specific activity, 22.9 Ci/mmol; PerkinElmer), 70 nmol [^3^H]progesterone (specific activity, 96.6 Ci/mmol; PerkinElmer), 70 nmol [^3^H]androstenedione (specific activity, 98.2 Ci/mmol; PerkinElmer), or 70 nmol [^3^H]testosterone (specific activity, 70 Ci/mmol; PerkinElmer) for 0 or 6 h at 37 °C in a water-saturated atmosphere (5% CO_2_, 95% air) to maintain the pH at 7.4. After incubation, steroids were extracted with ethyl acetate and subjected to HPLC analysis using a reversed-phase column, LiChrospher 100 RP-18 (Kanto Kagaku). Tritiated steroids (7α-hydroxypregnenolone, progesterone, 5α-dihydroprogesterone, androstenedione, testosterone, 5α-dihydrotestosterone, estradiol-17β, and cortisol) were chromatographed as standards to detect their elution positions. To confirm the involvement of steroidogenic enzymes in the formation of steroids, tritiated steroids were cultured with 50 μM ketoconazole (Sigma-Aldrich, St. Louis, MO, USA), an inhibitor of cytochrome P450s^13,26,28^; 50 μM trilostane (Sigma-Aldrich), an inhibitor of 3β-HSDs^25,26^; or 50 μM dutasteride (Sigma-Aldrich), an inhibitor of 5α-reductases^31^. All tritiated steroids were purchased from PerkinElmer.

### Quantification of steroidogenic enzyme activity

To compare the activities of steroidogenic enzymes, COS-7 cells were cultured in serum-free DMEM containing 70 nmol [^3^H]pregnenolone, 70 nmol [^3^H]progesterone, 70 nmol [^3^H]androstenedione, or 70 nmol [^3^H]testosterone for 6 h at 37 °C in a water-saturated atmosphere (5% CO_2_, 95% air) to maintain the pH at 7.4. After incubation, extracted steroids were subjected to HPLC analysis to measure the following metabolites: 3β-HSD product: progesterone; P4507α product: 7α-hydroxypregnenolone; P450c21 and P450c11β product: cortisol; 17β-HSD product: androstenedione; 5α-reductase product: 5α-dihydrotestosterone as described previously^13,25–30^. COS-7 cells were used in the experiments between passages 3 and 15, or passages 30 and 40.

### Statistical analysis

Data were statistically analyzed with a one-way ANOVA (when a normal distribution was found) and a *post hoc* Tukey-Kramer test. A significant difference was set at *P* < 0.05. All results were expressed as the mean ± SEM.

## Results

### Cholesterol does not convert to pregnenolone in COS-7 cells

**Figure 1:**
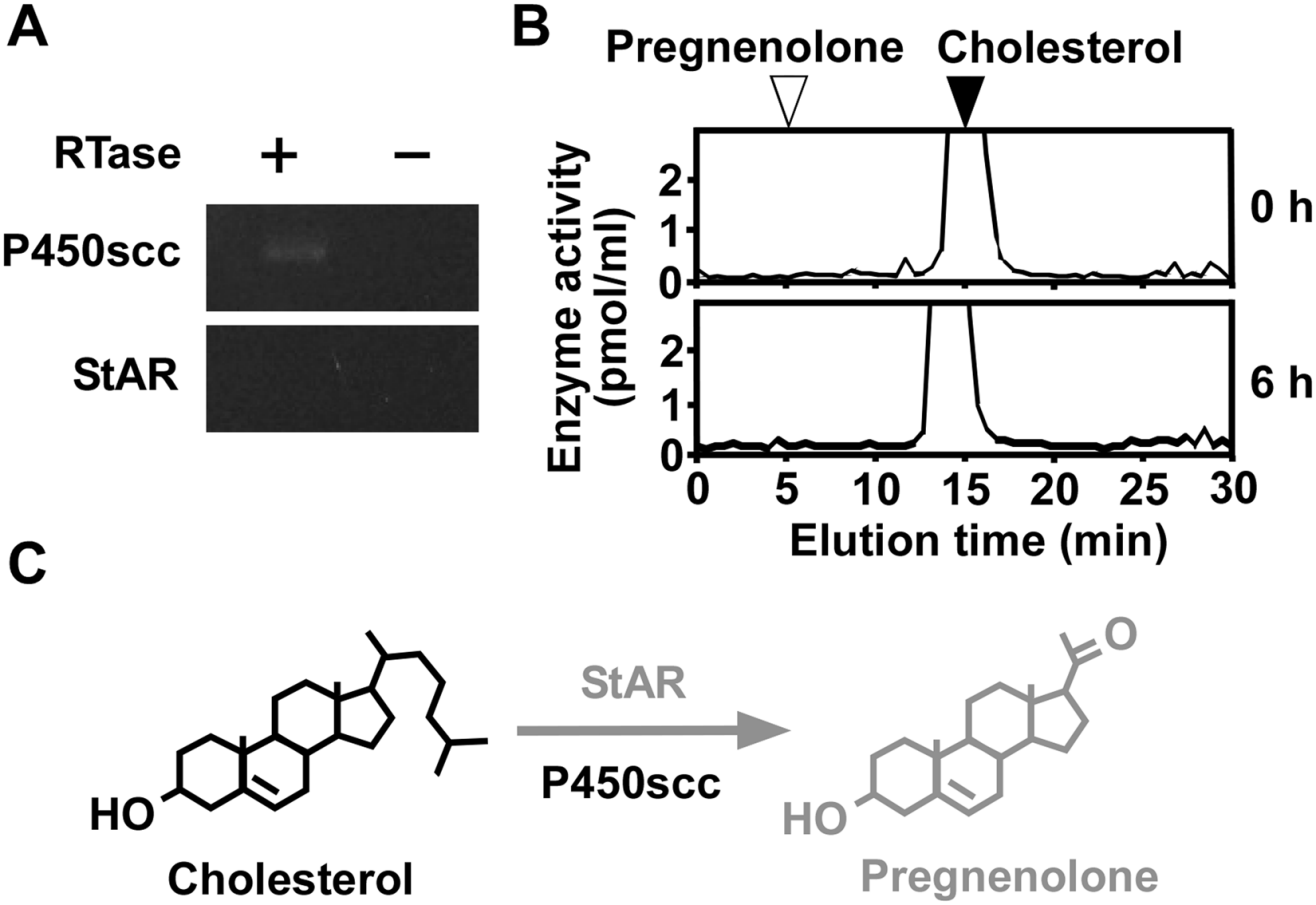
Cholesterol metabolism in COS-7 cells. (**A**) RT-PCR analyses of P450scc and StAR in COS-7 cells. Total RNA was extracted and reverse-transcribed with (+) or without (−) reverse transcriptase (RTase), followed by PCR amplification. (**B**) HPLC analysis of cholesterol metabolites in COS-7 cells. COS-7 cells were incubated with [^3^H]cholesterol and then each extract was analyzed using HPLC. The arrowheads indicate the elution positions of standard steroids, cholesterol, and pregnenolone. (**C**) Identified steroid biosynthetic pathways in COS-7 cells. Black font indicates confirmed steroidogenic enzymes or pathways. Gray font indicates steroidogenic enzymes or pathways that were not confirmed. Similar results were obtained in repeated experiments using three different samples.

To investigate the metabolism of cholesterol in COS-7 cells, RT-PCR analyses were used to detect the expression of steroidogenic and related enzymes, such as P450scc and StAR. RT-PCR analyses demonstrated the expression of P450scc mRNA, but not StAR mRNA in COS-7 cells (passages 3 to 15; Fig. 1A and Supplementary Fig. S1). Sequencing the amplified cDNA band verified that it was an authentic fragment of P450scc (GenBank accession no. XM_008015897).

To investigate cholesterol metabolism, COS-7 cells (passages 3 to 15) were incubated with [^3^H]cholesterol and the radioactive metabolites were analyzed by reversed-phase HPLC. As shown in Fig. 1B and C, no radioactive metabolites were detected.

### Pregnenolone is metabolized to progesterone and 7α-hydroxypregnenolone in COS-7 cells

**Figure 2:**
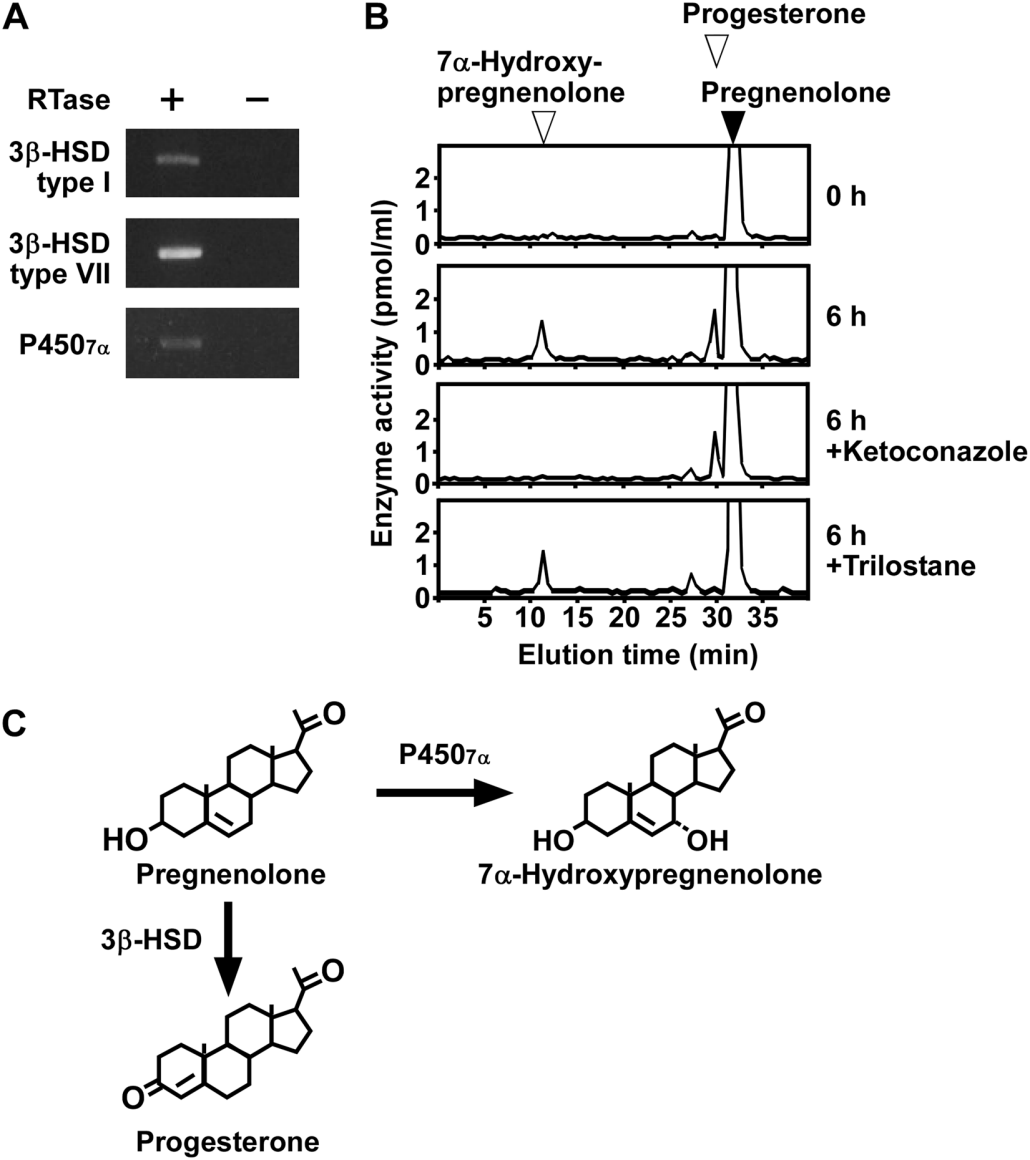
Steroid formation from pregnenolone in COS-7 cells. (**A**) RT-PCR analyses of steroidogenic enzymes 3β-HSD type I, 3β-HSD type VII, and P4507α in COS-7 cells. Total RNA was reverse-transcribed with (+) or without (−) RTase, followed by PCR amplification. (**B**) HPLC analyses of steroid formation in COS-7 cells. COS-7 cells were incubated with [^3^H]pregnenolone, and the extracts were analyzed using HPLC. COS-7 cells incubated with [^3^H]pregnenolone were also treated with ketoconazole, an inhibitor of P450s, or trilostane, an inhibitor of 3β-HSDs. The arrowheads indicate elution positions of standard steroids, pregnenolone (solid arrowhead), 7α-hydroxypregnenolone (open arrowhead), and progesterone (open arrowhead). (**C**) Identified steroid biosynthetic pathways in COS-7 cells. Black font indicates confirmed steroidogenic enzymes or pathways. Gray font indicates steroidogenic enzymes or pathways that were not confirmed. Similar results were obtained in repeated experiments using five different samples.

To investigate the metabolism of pregnenolone in COS-7 cells, RT-PCR analyses were performed to detect the expression of steroidogenic enzymes, such as 3β-HSD type I, 3β-HSD type VII, and P4507α. RT-PCR analyses demonstrated the expression of 3β-HSD type I, 3β-HSD type VII, and P4507α (passages 3 to 15; Fig. 2A and Supplementary Fig. S1). Sequencing of the amplified cDNA bands verified that they were authentic fragments of 3β-HSD type I (GenBank accession no. XM_007977369.1), 3β-HSD type VII (GenBank accession no. XM_007990382.1), and P4507α (GenBank accession no. XM_008000756.1).

To investigate steroid formation from pregnenolone, COS-7 cells (passages 3 to 15) were incubated with [^3^H]pregnenolone and the radioactive metabolites were analyzed by reversed-phase HPLC. Radioactive metabolites corresponding to 7α-hydroxypregnenolone and progesterone were detected (Fig. 2B,C). The concentration of these metabolites was reduced by treatment with ketoconazole, an inhibitor of P450s (Fig. 2B), and by trilostane, an inhibitor of 3β-HSDs (Fig. 2B).

### Progesterone is metabolized to 5α-dihydroprogesterone, cortisol, and androstenedione in COS-7 cells

**Figure 3:**
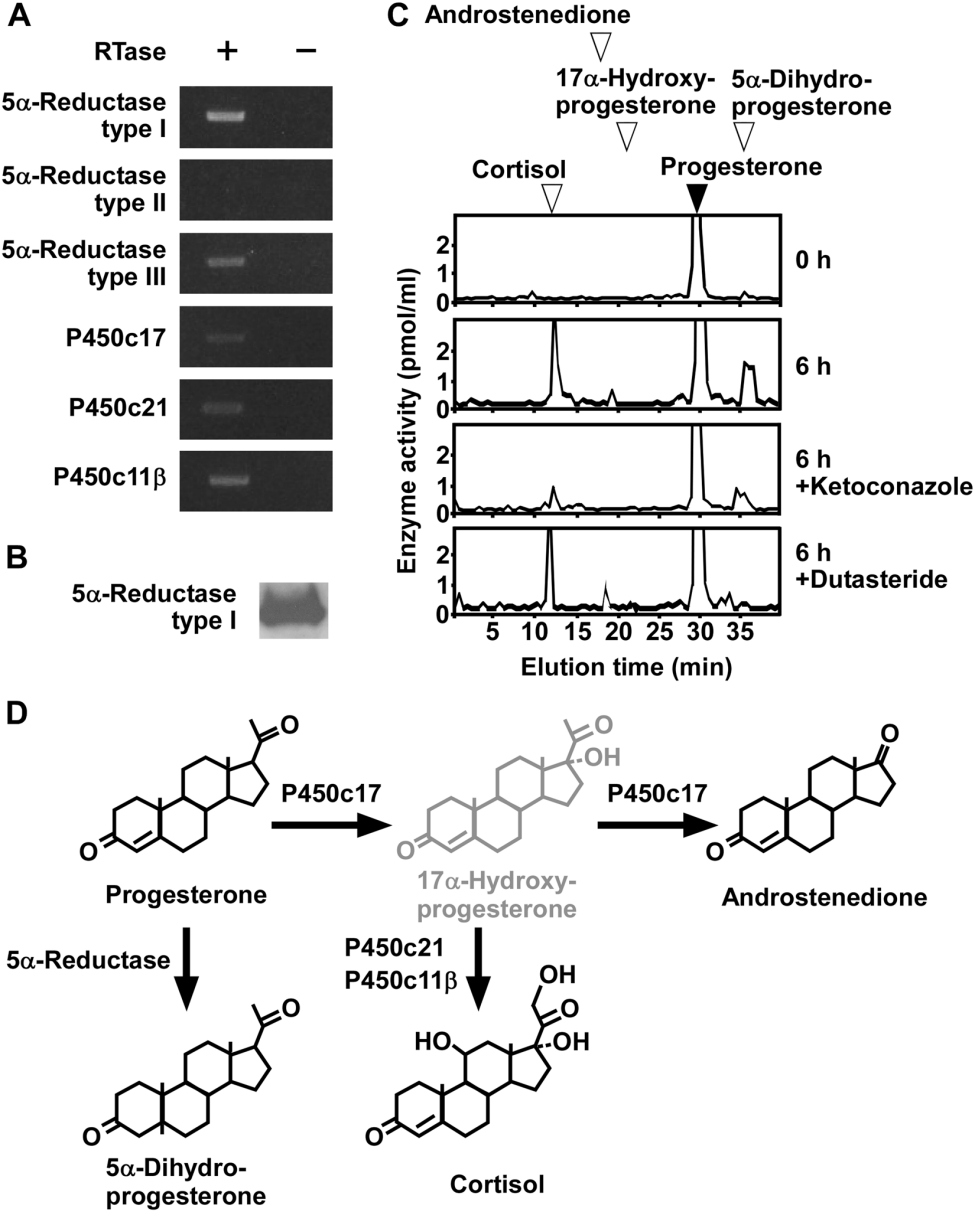
Steroid formation from progesterone in COS-7 cells. (**A**) RT-PCR analyses of steroidogenic enzymes 5α-reductase type I, 5α-reductase type II, 5α-reductase type III, P450c17, P450c21, and P450c11β in COS-7 cells. Total RNA was reverse-transcribed with (+) or without (−) RTase, followed by PCR amplification. (**B**) Western blot analysis of the steroidogenic enzyme 5α-reductase type I in COS-7 cells. (**C**) HPLC analyses of steroid formation in COS-7 cells. COS-7 cells were incubated with [^3^H]progesterone, and the extracts were analyzed using HPLC. COS-7 cells incubated with [^3^H]progesterone were also treated with ketoconazole, an inhibitor of P450s, or dutasteride, an inhibitor of 5α-reductases. The arrowheads indicate elution positions of standard steroids, progesterone (solid arrowhead), cortisol (open arrowhead), 17α-hydroxyprogesterone (open arrowhead), androstenedione (open arrowhead), and 5α-dihydroprogesterone (open arrowhead). (**D**) Identified biosynthetic pathways of steroids in COS-7 cells. Black font indicates confirmed steroidogenic enzymes or pathways. Gray font indicates steroidogenic enzymes or pathways that were not confirmed. Similar results were obtained in repeated experiments using five different samples.

To investigate the metabolism of progesterone in COS-7 cells, RT-PCR analyses were performed to detect the expression of steroidogenic enzymes, such as 5α-reductase type I, 5α-reductase type II, 5α-reductase type III, P450c17, P450c21, and P450c11β. RT-PCR analyses demonstrated the expression of 5α-reductase type I, 5α-reductase type III, P450c17, P450c21, and P450c11β, but not 5α-reductase type II (passages 3 to 15; Fig. 3A and Supplementary Fig. S1). Sequencing the amplified cDNA bands verified that 5α-reductase type I (GenBank accession no. XM_007961172.1), 5α-reductase type III (GenBank accession no. XM_007998686.1), P450c17 (GenBank accession no. XM_007963991), P450c21 (GenBank accession no. XM_007973036.1), and P450c11β (GenBank accession no. XM_008001709.1) were expressed in COS-7 cells. In addition, western blot analysis demonstrated the expression of 5α-reductase type I protein in COS-7 cells (passages 3 to 15; Fig. 3B and Supplementary Fig. S2).

To investigate steroid formation from progesterone, COS-7 cells (passages 3 to 15) were incubated with [^3^H]progesterone and the radioactive metabolites were analyzed by reversed-phase HPLC. Radioactive metabolites corresponding to 5α-dihydroprogesterone, cortisol, and androstenedione were detected (Fig. 3C,D). The concentration of these metabolites was reduced by treatment with ketoconazole, an inhibitor of P450s (Fig. 3C), and by dutasteride, an inhibitor of 5α-reductases (Fig. 3C).

### Androstenedione is metabolized to 5α-dihydrotestosterone in COS-7 cells

**Figure 4:**
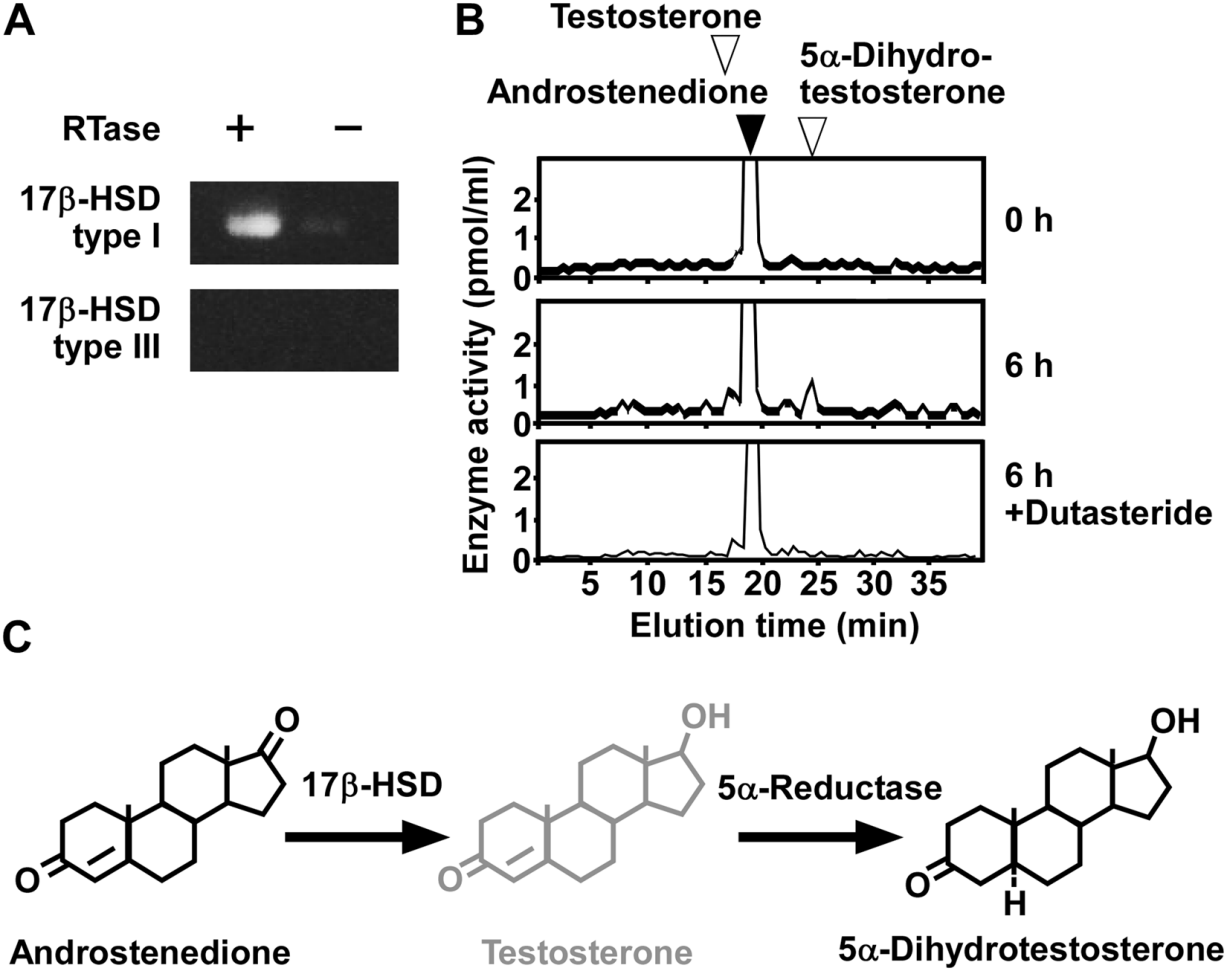
Steroid formation from androstenedione in COS-7 cells. (**A**) RT-PCR analyses of steroidogenic enzymes 17β-HSD type I and 17β-HSD type III in COS-7 cells. Total RNA was reverse-transcribed with (+) or without (−) RTase, followed by PCR amplification. (**B**) HPLC analyses of steroid formation in COS-7 cells. COS-7 cells were incubated with [^3^H]androstenedione and the extracts were analyzed using HPLC. COS-7 cells incubated with [^3^H]androstenedione were also treated with dutasteride, an inhibitor of 5α-reductases. The arrowheads indicate elution positions of standard steroids, androstenedione (solid arrowhead), testosterone (open arrowhead), and 5α-dihydrotestosterone (open arrowhead). (**C**) Identified biosynthetic pathways of steroids in COS-7 cells. Black font indicates confirmed steroidogenic enzymes or pathways. Gray font indicates steroidogenic enzymes or pathways that were not confirmed. Similar results were obtained in repeated experiments using five different samples.

To investigate the metabolism of androstenedione in COS-7 cells (passages 3 to 15), RT-PCR analyses were performed to detect the expression of 17β-HSD type I and 17β-HSD type III. RT-PCR analyses demonstrated the expression of 17β-HSD type I, but not 17β-HSD type III (Fig. 4A and Supplementary Fig. S1). Sequencing the amplified cDNA band verified that it was an authentic fragment of 17β-HSD type I.

To investigate steroid formation from androstenedione, COS-7 cells (passages 3 to 15) were incubated with [^3^H]androstenedione and the radioactive metabolites were analyzed by reversed-phase HPLC. A radioactive metabolite corresponding to 5α-dihydrotestosterone was detected (Fig. 4B,C), but testosterone, a precursor of 5α-dihydrotestosterone, was not detected (Fig. 4B,C).

### Testosterone is metabolized to androstenedione and 5α-dihydrotestosterone in COS-7 cells

**Figure 5:**
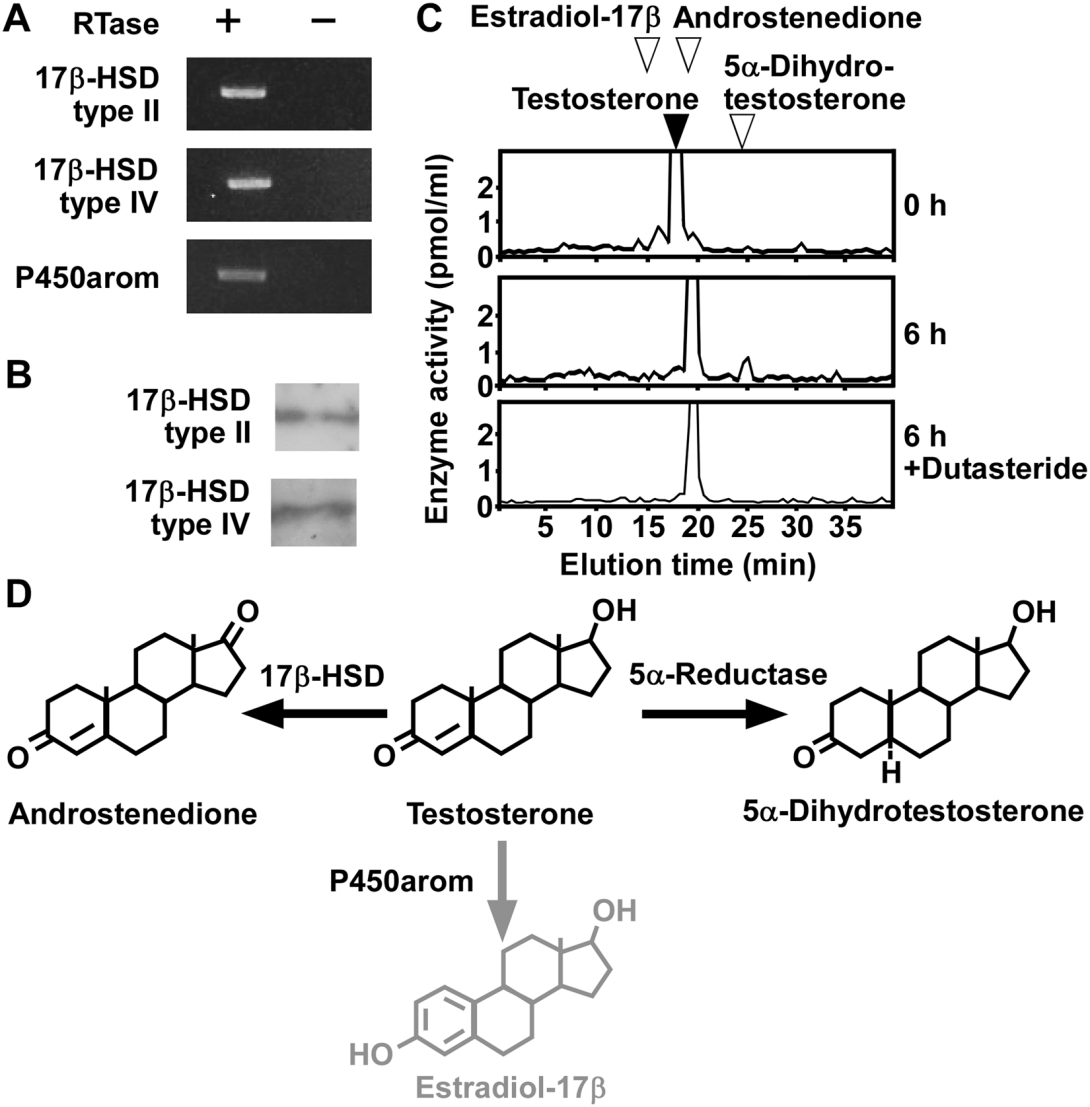
Steroid formation from testosterone in COS-7 cells. (**A**) RT-PCR analyses of steroidogenic enzyme 17β-HSD type II, 17β-HSD type IV, and P450arom in COS-7 cells. Total RNA was reverse-transcribed with (+) or without (−) RTase, followed by PCR amplification. (**B**) Western blot analyses of steroidogenic enzymes 17β-HSD type II and 17β-HSD type IV in COS-7 cells. (**C**) HPLC analyses of steroid formation in COS-7 cells. COS-7 cells were incubated with [^3^H]testosterone and the extracts were analyzed using HPLC. COS-7 cells incubated with [^3^H]testosterone were also treated with dutasteride, an inhibitor of 5α-reductases. The arrowheads indicate elution positions of standard steroids, testosterone (solid arrowhead), estradiol-17β (open arrowhead), androstenedione (open arrowhead), and 5α-dihydrotestosterone (open arrowhead). (**D**) Identified biosynthetic pathways of steroids in COS-7 cells. Black font indicates confirmed steroidogenic enzymes or pathways. Gray font indicates steroidogenic enzymes or pathways that were not confirmed. Similar results were obtained in repeated experiments using five different samples.

To investigate the metabolism of testosterone in COS-7 cells (passages 3 to 15), RT-PCR analyses were performed to detect the expression of 17β-HSD type II, 17β-HSD type IV, and P450arom. RT-PCR analyses demonstrated the expression of 17β-HSD type II, 17β-HSD type IV, and P450arom (Fig. 5A and Supplementary Fig. S1). Sequencing the amplified cDNA bands verified that they were authentic fragments of 17β-HSD type II (GenBank accession no. XM_007994189.1), 17β-HSD type IV (GenBank accession no. XM_008014219.1), and P450arom (GenBank accession no. XM_008016613.1). In addition, western blot analysis demonstrated the expression of 17β-HSD type II and 17β-HSD type IV proteins in COS-7 cells (passages 3 to 15; Fig. 5B and Supplementary Fig. S2).

To investigate steroid formation from testosterone, COS-7 cells (passages 3 to 15) were incubated with [^3^H]testosterone and the radioactive metabolites were analyzed by reversed-phase HPLC. Radioactive metabolites corresponding to androstenedione and 5α-dihydrotestosterone were detected (Fig. 5C,D). The 5α-dihydrotestosterone peak was reduced after treatment with dutasteride, an inhibitor of 5α-reductases (Fig. 5C). Estradiol-17β, a metabolite of testosterone, was not detected (Fig. 5C,D).

### Identified steroidogenic pathways and comparison of steroidogenic enzyme activities in COS-7 cells

**Figure 6:**
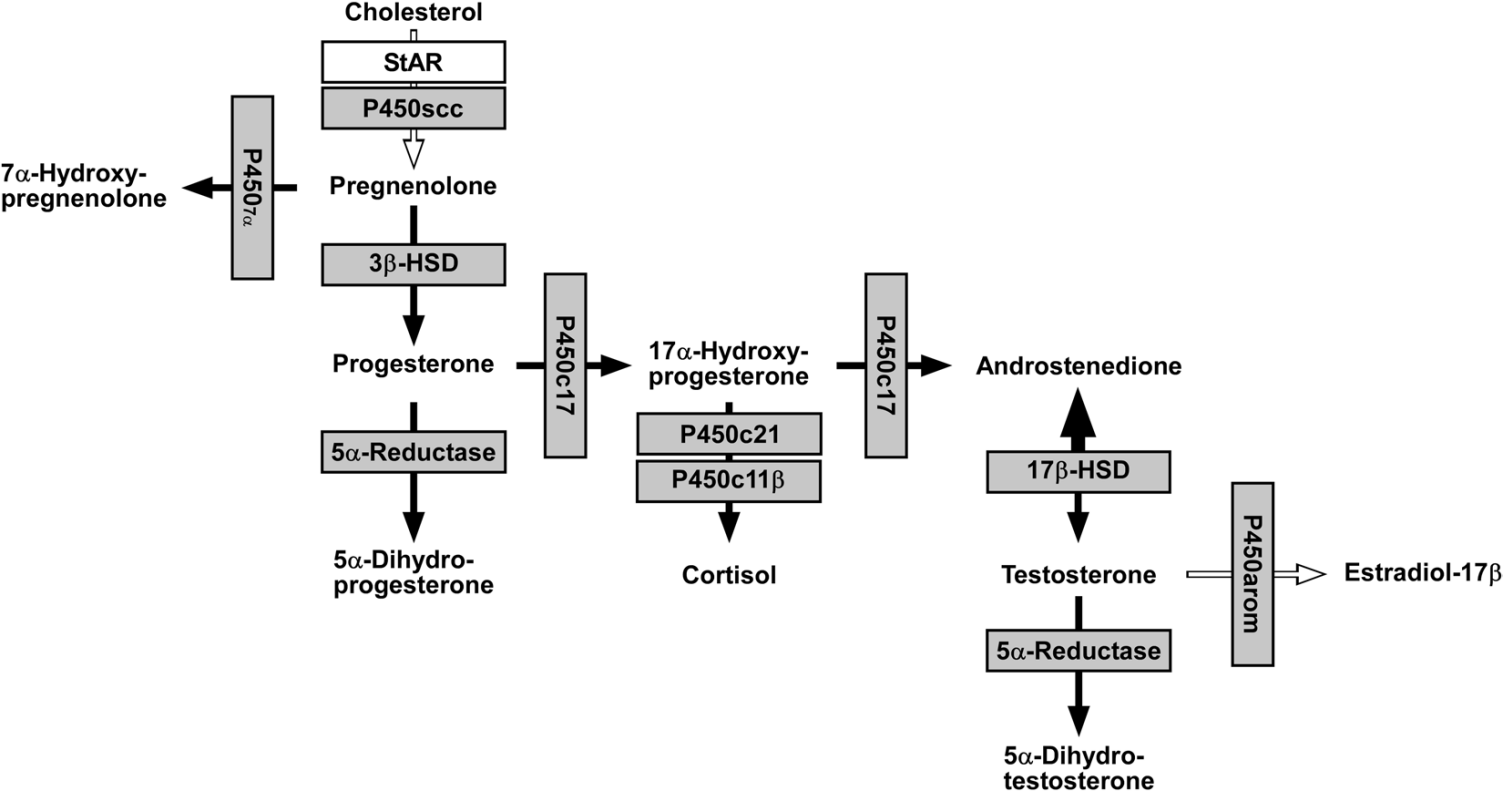
Identified biosynthetic pathways of steroids in COS-7 cells. COS-7 cells express P450scc, 3β-HSDs, P4507α, P450c17, 17β-HSDs, 5α-reductases, P450c21, P450c11β, and P450arom. In addition, steroidogenic enzymes 3β-HSD, P4507α, 5α-reductase, P450c17, P450c21, P450c11β, and 17β-HSD were active in cultured COS-7 cells. Gray boxes indicate confirmed expression of mRNAs. White box indicates mRNAs that were not confirmed. Black arrows indicate confirmed activity of steroidogenic enzymes. White arrows indicate steroidogenic enzymes that were not confirmed.

**Figure 7:**
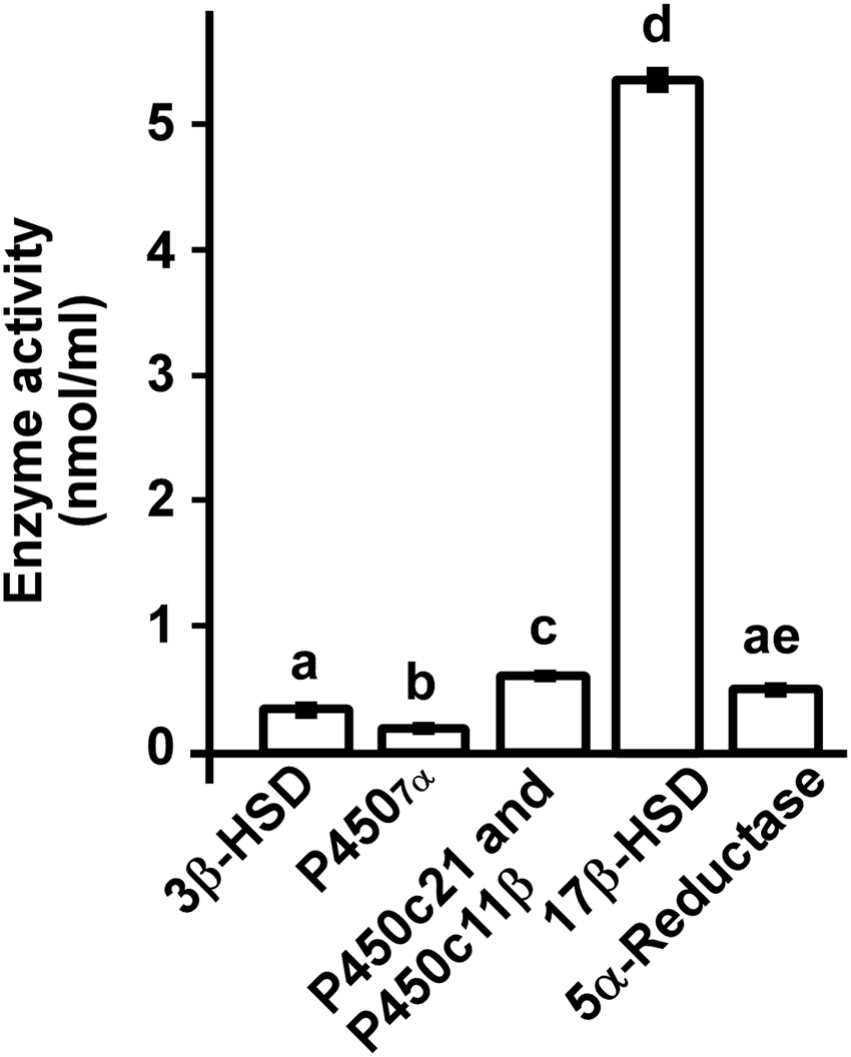
Comparison of the activities of steroidogenic enzymes in COS-7 cells. The activity of steroidogenic enzymes were analyzed in COS-7 cells. COS-7 cells were cultured in serum-free DMEM containing 70 nmol [^3^H]pregnenolone, 70 nmol [^3^H]17α-hydroxyprogesterone, or 70 nmol [^3^H]testosterone for 6 h. After incubation, the extracted steroids were subjected to HPLC analysis to measure the metabolites (3β-HSD product: progesterone; P4507α product: 7α-hydroxypregnenolone; P450c21 and P450c11β product: cortisol; 17β-HSD product: androstenedione; 5α-reductase product: 5α-dihydrotestosterone).

Thus, the active steroidogenic enzymes in cultured COS-7 cells were identified as 3β-HSD, P4507α, 5α-reductase, P450c17, P450c21, P450c11β, and 17β-HSD (Fig. 6). To compare the activities of steroidogenic enzymes in COS-7 cells (passages 3 to 15), we analyzed their metabolites by reversed-phase HPLC. COS-7 cells were cultured in serum-free DMEM containing 70 nmol [^3^H]pregnenolone, 70 nmol [^3^H]17α-hydroxyprogesterone, or 70 nmol [^3^H]testosterone for 6 h. After incubation, the extracted steroids were subjected to HPLC analyses to measure metabolites. The activity of 17β-HSD that metabolites testosterone to androstenedione was significantly higher than those of other steroidogenic enzymes in COS-7 cells (Fig. 7).

To investigate the effect of molecules in FBS on steroid formation, COS-7 cells (passages 3 to 15) were cultured in 10%FBS supplemented DMEM or FBS-free DMEM to 90% confluence. At 90% confluence, COS-7 cells were cultured in serum-free DMEM containing 70 nmol [^3^H]pregnenolone, 70 nmol [^3^H]17α-hydroxyprogesterone, or 70 nmol [^3^H]testosterone for 6 h. After incubation, the extracted steroids were subjected to HPLC analyses to measure metabolites. The absence of FBS did not alter the activity of steroidogenic enzymes significantly in COS-7 cells (Supplementary Fig. S3).

In addition, the number of passages of the cells is an important factor for their function. Therefore, the activities of steroidogenic enzymes in cultured COS-7 cells were measured. In COS-7 cells between passages 30 and 40, the enzymatic activities of 3β-HSD and 5α-reductase were increased compared to those of COS-7 cells between passages 3 and 15 (Supplementary Fig. S4).

## Discussion

COS-7 cells are derived from the kidney, which is known as a non-steroidogenic organ^9,10^. Therefore, COS-7 cells were also generally considered a non-steroidogenic cell line^6–8^. However, the present study demonstrates that COS-7 cells express a series of steroidogenic enzymes and metabolize a variety of steroids. We first demonstrated that the mRNAs of P450scc, 3β-HSDs, P4507α, P450c17, 17β-HSDs, 5α-reductases, P450c21, P450c11β, and P450arom are expressed in COS-7 cells. In addition, steroidogenic enzymes 3β-HSD, P4507α, 5α-reductase, P450c17, P450c21, P450c11β, and 17β-HSD were active in cultured COS-7 cells.

The kidney is generally considered to be a non-steroidogenic organ^9,10^; however, steroidogenesis in kidney tissue has been reported by some groups. Expression of P450c11β protein has been shown by western blotting and immunohistochemistry in normal human kidney^19^. In addition, northern blot analysis has detected 17β-HSD type XI mRNA expression in the human kidney^21^. In male and female rat kidneys, P450scc^24^ and 3β-HSDs^22^ mRNAs and 3β-HSD protein^22^ have been detected. The expression of P450scc in the rat kidney localizes to the cortical distal tubules and is high during the first days of life^23,24^. According to Valle *et al*., [^14^C]progesterone is metabolized to 5α-dihydroprogesterone, 11-deoxycorticosterone, 17α-hydroxyprogesterone, androstenedione, and testosterone in the kidney tissue, from rats of both sexes^23,24^. These previous studies suggest that the kidney has the ability for local steroid production. These reports also support our findings of steroid metabolism in COS-7 cells.

In this study, we found that 5α-dihydrotestosterone, a metabolite of testosterone, was produced from androstenedione, a precursor of testosterone, in COS-7 cells. However, we could not detect a testosterone peak by reversed-phase HPLC in this system (Fig. 4B). Furthermore, testosterone was depleted from the medium after 6 h of incubation (Fig. 5B). Our results suggest that the activity of 17β-HSD type II and IV is higher than that of 17β-HSD type I in COS-7 cells (Fig. 6). In addition, a previous study has shown that the activity of 5α-reductase, an enzyme that converts testosterone into dihydrotestosterone, is high in the kidney^23,24^. Furthermore, testosterone is produced in the rat kidney^23,24^. These results suggest that testosterone is produced from androstenedione in COS-7 cells, but the activities of 17β-HSD type II and IV and 5α-reductase were high enough to deplete testosterone from COS-7 cell culture media. However, we could not detect estradiol-17β in cultured COS-7 cells after the incubation with testosterone although P450arom are expressed in COS-7 cells (Fig. 5). It is considered that COS-7 cells may not convert androgen to estrogen. Further studies are needed to confirm this conclusion.

COS-7 cells are used for transfection experiments to analyze the function of steroidogenic genes^11–13^, the function of steroid receptors^14–16^, and the effects of steroids on functional molecules^17,18^. However, our findings here suggest that testosterone is actively converted to androstenedione or 5α-dihydrotestosterone in COS-7 cells. Thus, it should be noted that COS-7 cells are unfit to use for analyzing the effects of testosterone by testosterone addition *in vitro*. Past researchers probably inferred from their experience that testosterone is inactivated in COS-7 cells. In fact, they used synthetic, non-metabolizable androgen instead of testosterone in COS-7 cells^32,33^.

In the present study, steroidogenic enzymes 3β-HSD, P4507α, 5α-reductase, P450c17, P450c21, P450c11β, and 17β-HSD were active in cultured COS-7 cells. However, as shown in Fig. 7, the steroidogenic enzyme activities of 3β-HSD, P4507α, 5α-reductase, P450c21, and P450c11β were low compared to the activity of 17β-HSD in cultured COS-7 cells. Thus, the activities of these enzymes might not influence the analysis of the function of steroidogenic genes. In fact, in a previous study^30^, we failed to detect enzymatic activity of P4507α in COS-7 cells. This discrepancy may be due to differences in the cell cycle stage of COS-7 cells. A future study is needed to confirm this hypothesis.

## Electronic supplementary material

Supplementary Information
