# Supplementary material for: Expression of steroidogenic enzymes and metabolism of steroids in COS-7 cells known as non-steroidogenic cells
Source: Sci Rep. 2018 Feb 1;8:2167. doi: 10.1038/s41598-018-20226-2 (PMC5794755; doi:10.1038/s41598-018-20226-2)
Supplement: Supplementary file 1 — Supplementary Information [file 41598_2018_20226_MOESM1_ESM.pdf]

Expression of steroidogenic enzymes and metabolism of steroids in COS-7 cells known as non-steroidogenic cells

Mitsuki Nozaki[1], Shogo Haraguchi[1,2,*], Takuro Miyazaki[2], Daichi Shigeta[1], Noriko Kano[1], Xiao-Feng Lei[2], Joo-ri Kim-Kaneyama[2], Hiroyuki Minakata[3], Akira Miyazaki[2] & Kazuyoshi Tsutsui[1,*]

[1]Laboratory of Integrative Brain Sciences, Department of Biology and Center for Advanced Biomedical Sciences of Waseda University, Tokyo 162-8480, Japan

[2]Department of Biochemistry, Showa University School of Medicine, Tokyo 142-8555, Japan

[3]Suntory Foundation for Life Sciences, Kyoto 619-0284, Japan

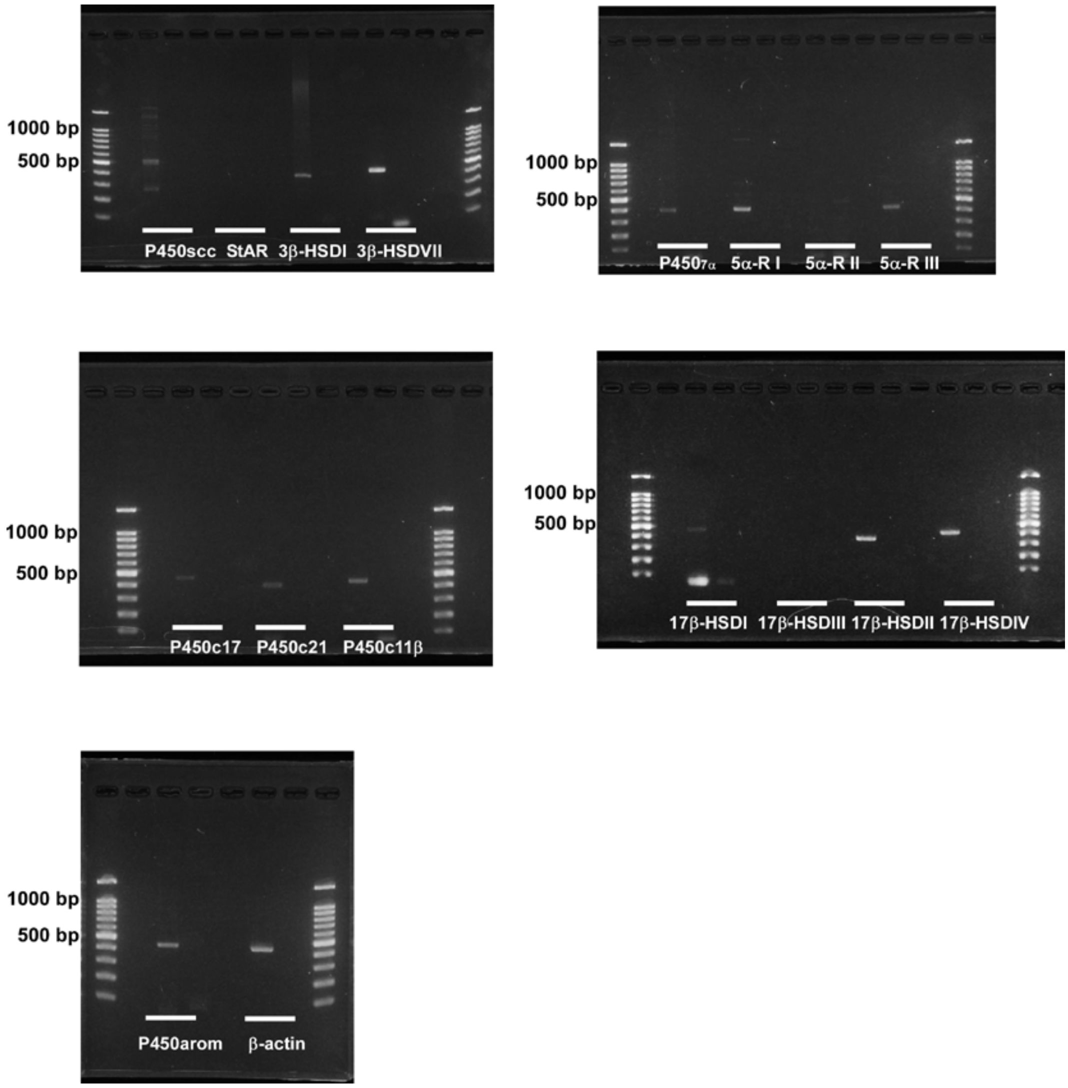

Supplementary Figure S1. Uncropped scans of RT-PCR.

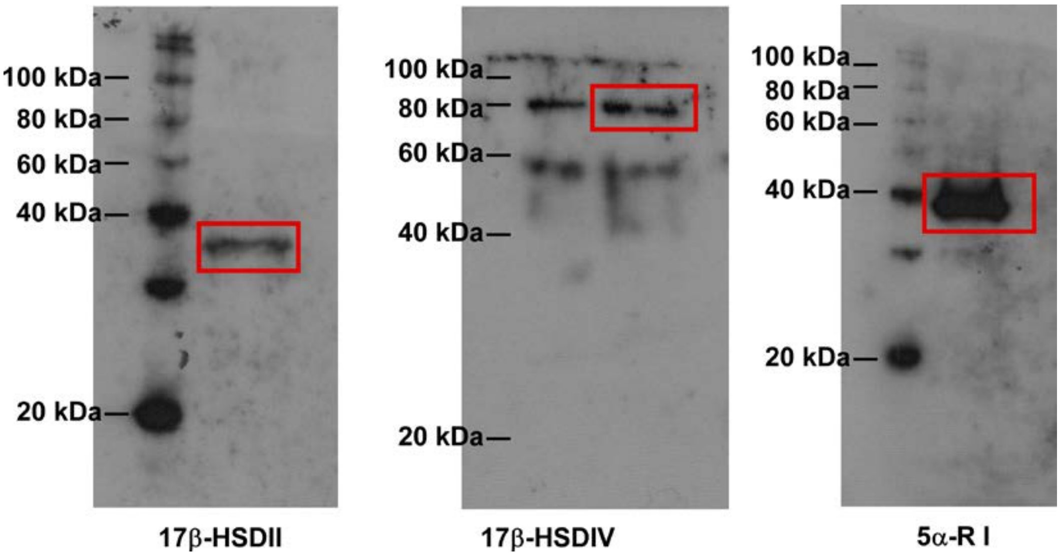

Supplementary Figure S2. Uncropped scans of Western blots.

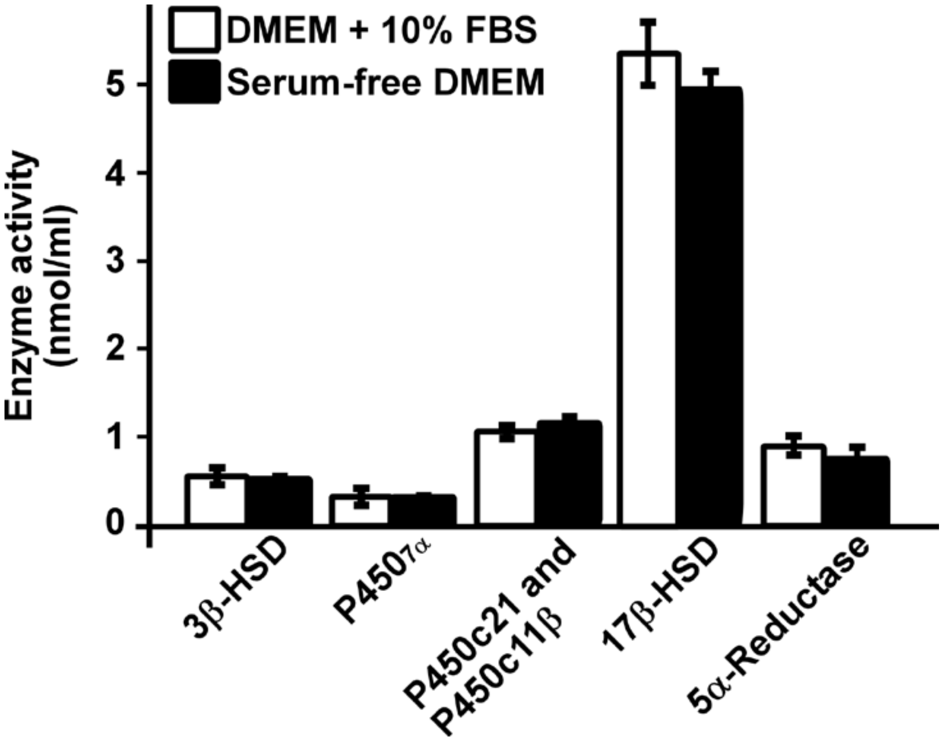

Supplementary Figure S3. The absence of FBS did not alter the activity of steroidogenic enzymes significantly in COS-7 cells.

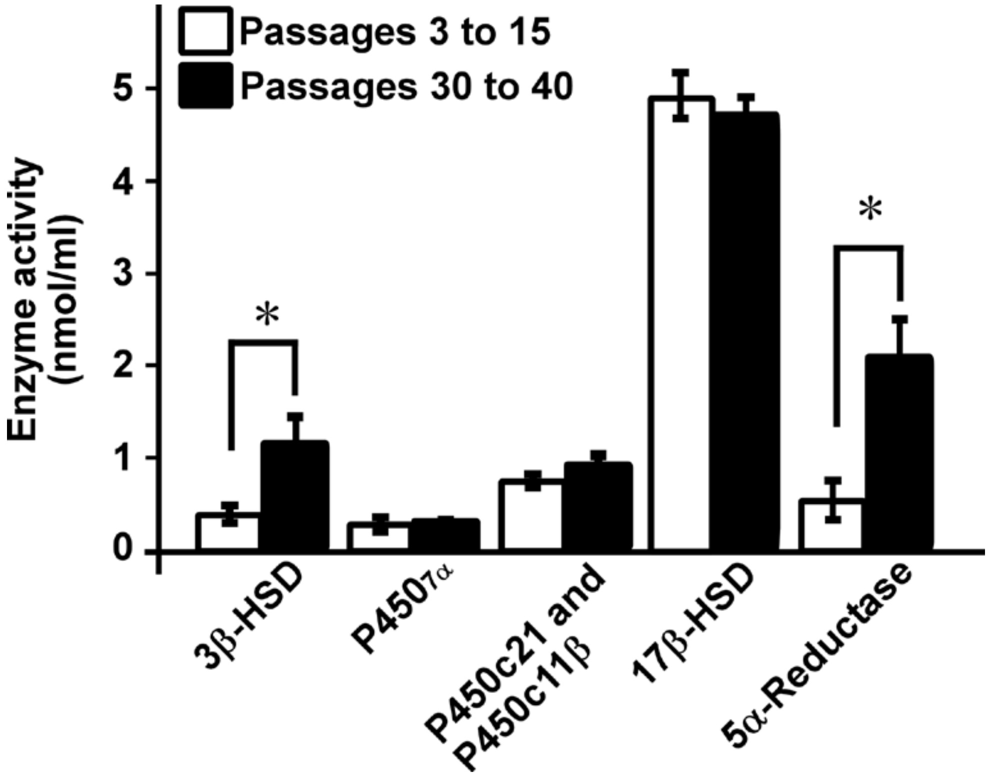

Supplementary Figure S4. In COS-7 cells between passages 30 and 40, the enzymatic activities of 3β-HSD and 5α-reductase were increased compared to those of COS-7 cells between passages 3 and 15. Each column and vertical line represent the mean ± s.e.m. $^*P < 0.05$.
